# The Ministry and Its Ministers

**Published:** 1923-11

**Authors:** M. W. Maxwell

**Affiliations:** Yelton Hotel, Hastings


### CORRESPONDENCE.

[Correspondence on all subjects is invited, but we cannot be responsible for the opinions expressed by our correspondents, who must give name and address as a guarantee of good faith, but not necessarily for publication. Correspondents are reminded that conciseness greatly facilitates early insertion.]

### The Ministry and Its Ministers.

*To the Editor of* THE HOSPITAL AND HEALTH REVIEW.

SIR,—I have read with much interest your article on the need for a permanent Health Minister. It seems to me that you have drawn attention to a most vital necessity. It is true that we over here are not in quite such a deplorable condition as America, which suffers a complete change of all Government officials, even down to the postmaster of the smallest village, with each change of administration; but even so, we are surely bad enough!

It is understandable and reasonable enough that the Ministries dealing with such matters as Foreign Affairs, the Colonies, Trade, or any other department whose policy must necessarily depend on the general policy of the Government at the moment in power, should be controlled by members of that Government; but in a Ministry which is—or should be—solely concerned with social welfare there is no such necessity. The head of such a department should be as secure of office, as free from political bids as is, by tradition, the Speaker. The only qualifications necessary should be efficiency and a thorough understanding of the work to be done.

M. W. MAXWELL.

Yelton Hotel,
Hastings.

